# Bilateral internal carotid artery fibromuscular dysplasia identified during acute ischemic stroke evaluation: A case report

**DOI:** 10.1016/j.radcr.2026.06.154

**Published:** 2026-07-31

**Authors:** Wejdan Hashim AlMusallam, Aqeel Alhashim, Noora Adam Alabdulmohsen

**Affiliations:** a Radiology Department, AlMoosa Health Group, Ahsaa, Saudi Arabia; b Department of Clinical Pharmacy, Imam Abdulrahman Bin Faisal University, Dammam, Saudi Arabia

**Keywords:** Bilateral, Internal carotid artery, Fibromuscular dysplasia

## Abstract

A 52-year-old woman with a known history of hypertension presented to the emergency department with acute confusion. She also had a prior history of transient ischemic attack. Computed tomography (CT) of the brain demonstrated an acute infarction involving the left insular cortex. CT angiography revealed no evidence of large-vessel occlusion; however, both cervical segments of the internal carotid arteries exhibited a tortuous course with a characteristic “string-of-beads” appearance, raising the possibility of incidental fibromuscular dysplasia (FMD). This finding was not considered the primary etiology of the acute infarction. The diagnosis of bilateral internal carotid artery fibromuscular dysplasia was subsequently confirmed by catheter angiography.

## Introduction

Fibromuscular dysplasia (FMD) is a noninflammatory, nonatherosclerotic arterial disease characterized by abnormal cellular proliferation and distortion of the arterial wall [1]. It most commonly affects the renal and carotid arteries, although involvement of other vascular territories has also been reported [2]. The etiology of FMD remains unclear. Several hypotheses regarding its pathogenesis have been proposed, including hormonal, mechanical, genetic, and ischemic mechanisms affecting the arterial wall [3,4].

Historically, FMD has been classified according to the arterial wall layer involved, namely the intima, media, or adventitia (periarterial layer) [5,6]. However, histopathological confirmation is not routinely required, as the diagnosis is primarily based on characteristic angiographic findings [7,8].

Cerebrovascular involvement of FMD was first confirmed histopathologically in 1965 in a patient with carotid artery disease [9]. In most cases, cerebrovascular FMD is detected incidentally during imaging studies performed for unrelated indications. In other instances, patients may present with manifestations such as ischemic or hemorrhagic stroke, transient ischemic attack, amaurosis fugax, or syncope [10,11].

We present the case of a 52-year-old woman who presented with an acute cerebral infarction and was incidentally found to have bilateral fibromuscular dysplasia involving the cervical segments of the internal carotid arteries. The FMD was considered an incidental finding and was not regarded as the primary etiology of the acute infarction.

### Case

A 52-year-old woman with a known history of hypertension and hypothyroidism presented to the emergency department with intermittent episodes of acute confusion. According to her family, the symptoms were first noticed at approximately 4:00 PM, when she was unable to answer questions appropriately and could not recall the names of family members. There was no history of fever or recent trauma. The patient reported experiencing a headache earlier that morning, which was relieved by paracetamol. She denied any focal neurological deficits, visual disturbances, gastrointestinal symptoms, or genitourinary complaints.

Her medical history was significant for a previous transient ischemic attack, for which she was taking aspirin (acetylsalicylic acid). Her home medications included amlodipine 5 mg once daily, atorvastatin 20 mg once daily, aspirin 81 mg once daily, levothyroxine 125 µg once daily, and iron and vitamin D supplements.

On physical examination, the patient was conscious, alert, and oriented to time, place, and person, with a Glasgow Coma Scale (GCS) score of 15/15. She was able to answer questions appropriately.

Vital signs were within normal limits. The patient was afebrile, hemodynamically stable, and breathing comfortably on room air. Neurological examination revealed a symmetrical face with no facial droop, mouth deviation, or dysarthria. Extraocular movements were intact, with no evidence of nystagmus. Visual fields were full to confrontation. There was no upper or lower limb drift, and muscle strength was 5/5 in all extremities. Sensory examination was unremarkable. The National Institutes of Health Stroke Scale (NIHSS) score ranged from 0 to 1, attributable primarily to a mild language deficit.

Laboratory investigations demonstrated a hemoglobin level of 8.1 g/dL, glycated hemoglobin (HbA1c) of 6.2%, and a low-density lipoprotein (LDL) cholesterol level of 2.5 mmol/L.

### Imaging findings

**Fig. 1:**
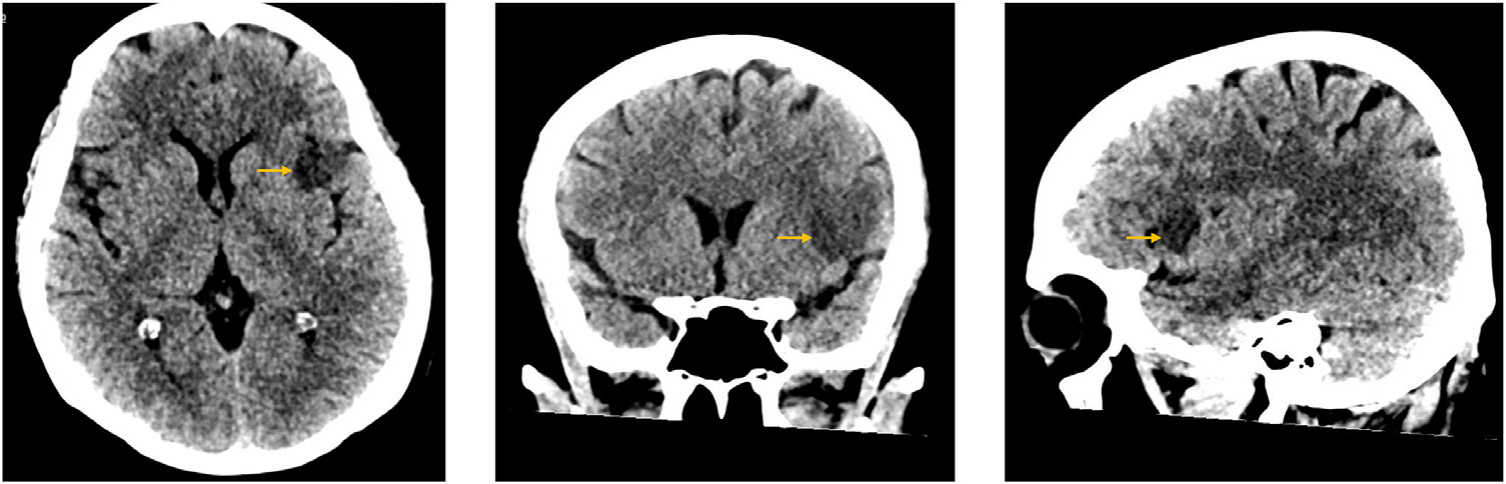
Computed tomography brain (axial, coronal and sagittal views) shows anterior aspect of the left insular cortex focal hypodensity with loss of grey-white matter differentiation and sulcal effacement, suggesting acute infarction.

Noncontrast computed tomography (CT) of the brain demonstrated a focal hypodense area involving the anterior aspect of the left insular cortex, associated with loss of gray-white matter differentiation and mild sulcal effacement, consistent with an acute infarction (Fig. 1).

**Fig. 2:**
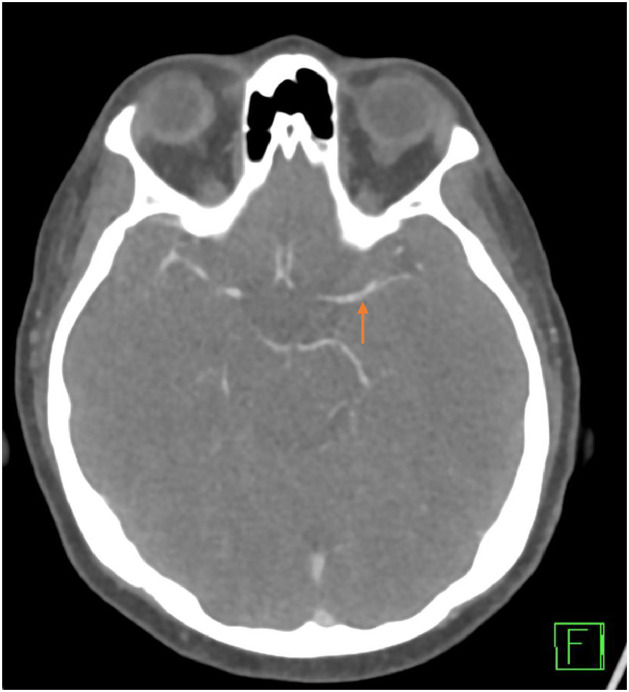
Single selected axial CTA shows the middle cerebral artery not occluded by thrombus or stenosis.

**Fig. 3:**
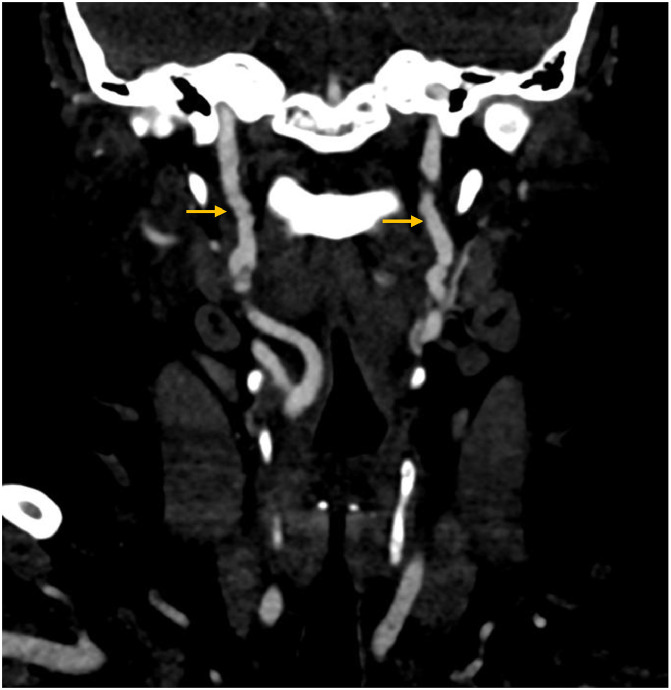
Computed tomography angiography coronal reformates, shows both internal carotid arteries are tortuous giving the string of beads appearance. No vessel occlusion.

Computed tomography angiography (CTA) revealed an acute left insular watershed infarction without evidence of large-vessel occlusion involving the intracranial arteries, including the left middle cerebral artery (Fig. 2). In addition, both cervical segments of the internal carotid arteries exhibited a tortuous course with a characteristic “string-of-beads” appearance, raising suspicion for fibromuscular dysplasia (Fig. 3).

**Fig. 4:**
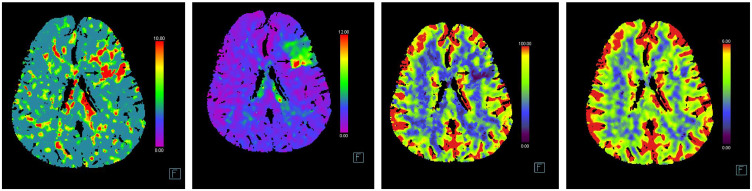
CT perfusion; from the left: Mean transit time (MTT), time-to-maximum (Tmax), cerebral blood flow (CBF), and cerebral blood volume (CBV), respectively. Study shows increased MTT, Tmax, and markedly decreased CBF, and normal CBV.

CT perfusion imaging demonstrated prolonged mean transit time (MTT) and time-to-maximum (Tmax), accompanied by markedly reduced cerebral blood flow (CBF), while cerebral blood volume (CBV) was preserved (Fig. 4).

**Fig. 5:**
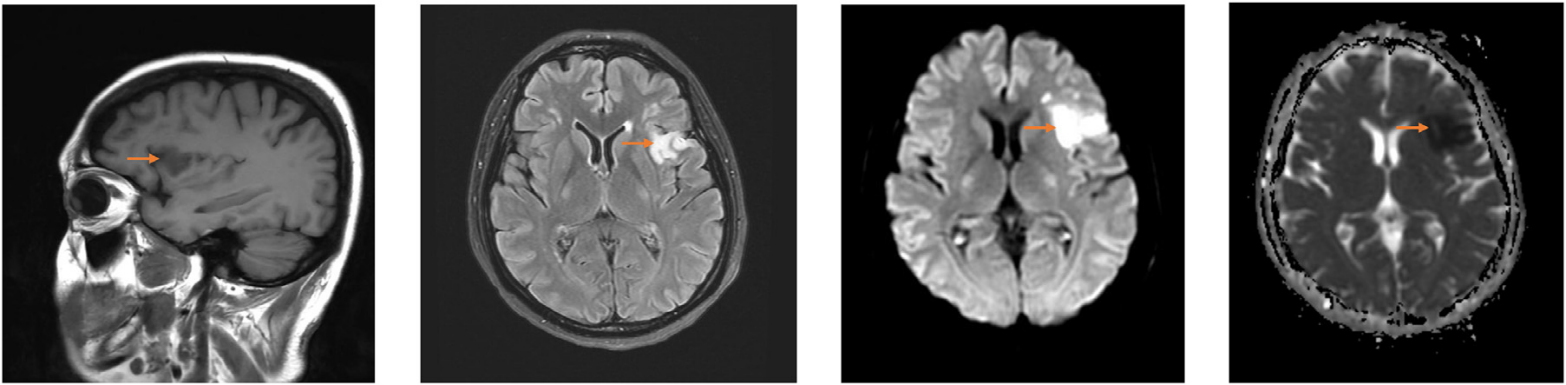
MRI brain from the left: T1-weighted image, T2 FLAIR weighted image, Diffusion-weighted image, and apparent diffusion coefficient map, respectively. Redemonstrations of the acute infarction in the left anterior insular cortex, low signal intensity in T1, high signal intensity on T2, and showing restrictive diffusion.

Magnetic resonance imaging (MRI) of the brain confirmed an acute infarction involving the left anterior insular cortex. The lesion demonstrated low signal intensity on T1-weighted images, high signal intensity on T2-weighted and fluid-attenuated inversion recovery (FLAIR) sequences, and restricted diffusion on diffusion-weighted imaging (DWI) (Fig. 5). No evidence of hemorrhagic transformation was identified.

**Fig. 6:**
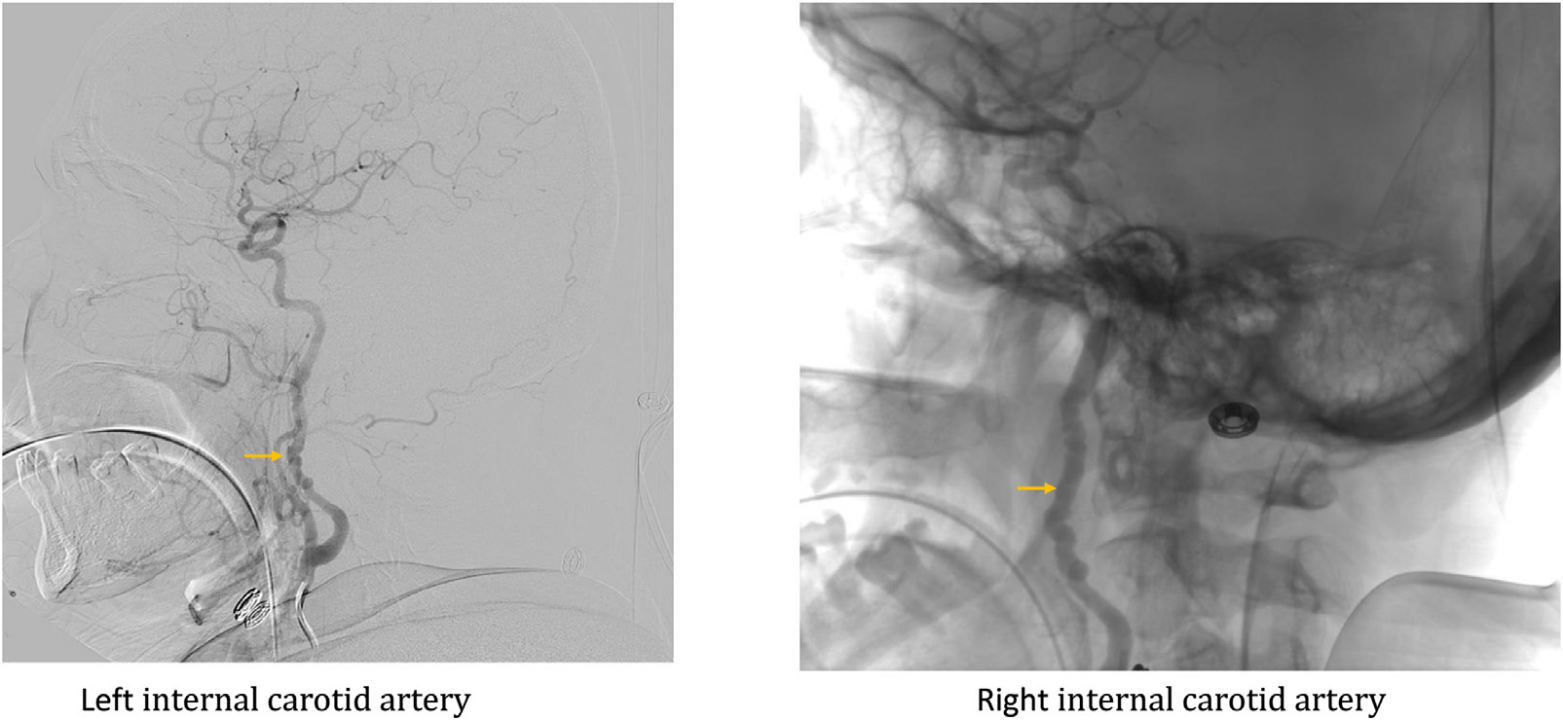
Digital subtraction angiography study lateral view of the right and left internal carotid arteries confirmed diagnosis of bilateral internal carotid artery fibromuscular dysplasia, without significant stenosis or occlusion.

Digital subtraction angiography (DSA) confirmed bilateral multifocal fibromuscular dysplasia involving the mid-to-distal cervical segments of the internal carotid arteries. The angiographic findings demonstrated alternating areas of stenosis and mild ectasia, producing the characteristic “string-of-beads” appearance (Fig. 6). The petrous and cavernous segments of both internal carotid arteries were preserved, with no significant involvement identified. No associated arterial dissection, pseudoaneurysm formation, or intraluminal thrombus was observed.

Additional vascular screening to assess involvement of other vascular territories was not performed, representing a limitation of this report. Given the systemic nature of fibromuscular dysplasia, comprehensive vascular evaluation is recommended to identify multifocal arterial involvement and associated vascular abnormalities.

The patient was managed conservatively with dual antiplatelet therapy (DAPT) consisting of ticagrelor and aspirin, initiated according to standard management protocols for minor ischemic stroke rather than specifically for fibromuscular dysplasia. Dual antiplatelet therapy was continued for 3 months, followed by transition to long-term single antiplatelet therapy for secondary stroke prevention.

No endovascular intervention was indicated, as there was no hemodynamically significant stenosis or other vascular abnormality requiring revascularization. Neurology follow-up was arranged for ongoing clinical and imaging surveillance. Follow-up CT of the brain demonstrated no new ischemic or hemorrhagic events.

Blood pressure monitoring and optimization were emphasized as essential components of long-term vascular risk reduction and the overall management of fibromuscular dysplasia.

## Discussion

Fibromuscular dysplasia (FMD) is a noninflammatory, nonatherosclerotic arterial disease that was first described in 1938 by Leadbetter and Burkland [1]. FMD most commonly affects the renal and carotid arteries; however, involvement of other vascular territories has also been reported, including the vertebral, iliac, subclavian, mesenteric, and peripheral arteries [2].

The etiology of FMD remains incompletely understood. Several hypotheses have been proposed to explain its pathogenesis. According to the literature [3,4], 4 major mechanisms have been suggested:

1. Hormonal hypothesis: The marked female predominance among affected individuals suggests that hormonal factors may contribute to disease development.
2. Mechanical hypothesis: A history of trauma or repeated microtrauma to the arterial wall has been proposed as a potential contributing factor, potentially predisposing individuals to the development of FMD.
3. Genetic hypothesis: Familial clustering has been reported, and some studies have identified affected family members within the same pedigree, suggesting a possible genetic predisposition.
4. Ischemic hypothesis: Ischemic injury to the vasa vasorum may result in structural and morphological changes within the arterial wall, contributing to the development of FMD.

Fibromuscular dysplasia (FMD) has traditionally been classified according to the arterial wall layer involved, namely the intima, media, or adventitia (periarterial layer) [5,6]. The media is the most commonly affected layer and is further subdivided into medial fibroplasia, perimedial fibroplasia, and medial hyperplasia. This histopathological classification was originally developed for renal artery involvement but is also applicable to FMD affecting other vascular territories [6,12].

Medial fibroplasia is the most common histological subtype of FMD. It is characterized by collagen deposition within areas of degenerating elastic fibers, resulting in fibromuscular thickening and rigidity of the arterial wall. These changes produce alternating segments of arterial stenosis and dilatation due to focal loss of smooth muscle cells. The resulting angiographic appearance is the classic “string-of-beads” pattern, which is considered highly characteristic of FMD. However, histopathological confirmation is not routinely required, as the diagnosis is primarily established based on characteristic angiographic findings [7,8].

Fibromuscular dysplasia (FMD) involving cerebral arteries was first histopathologically confirmed in 1965 in a case of carotid artery involvement [9]. In most cases, cerebrovascular FMD is detected incidentally during imaging performed for unrelated indications. In other cases, patients may present clinical manifestations such as ischemic or hemorrhagic stroke, transient ischemic attacks, amaurosis fugax, or syncope [10,11].

The literature indicates that the most common symptom associated with cerebrovascular FMD is chronic headache, most frequently of a migraine type. Migraine is a primary headache disorder characterized by recurrent episodes of moderate-to-severe headache, often accompanied by associated neurological and systemic symptoms [[13], [14], [15]]. The second most common symptom is pulsatile tinnitus, defined as the perception of rhythmic sound in the ear that is typically synchronous with the patient’s heartbeat. In contrast to idiopathic tinnitus, pulsatile tinnitus is more often associated with an identifiable underlying vascular etiology [7,16].

The diagnosis of fibromuscular dysplasia (FMD) is primarily based on imaging findings. Computed tomography angiography (CTA) and magnetic resonance angiography (MRA) typically demonstrate the characteristic “string-of-beads” appearance, resulting from alternating segments of arterial stenosis and dilatation [2,17].

Digital subtraction angiography (DSA) remains the reference standard for diagnosis; however, it is generally reserved for cases with inconclusive noninvasive imaging findings or when endovascular intervention is being considered.

Management of fibromuscular dysplasia (FMD) is guided by clinical presentation. Asymptomatic patients are generally managed conservatively with antiplatelet therapy and optimization of cardiovascular risk factors [2,17]. Endovascular intervention, including percutaneous transluminal angioplasty, is reserved for patients with recurrent ischemic symptoms or evidence of significant hemodynamic compromise [2].

Long-term clinical and imaging follow-up is recommended due to the potential risk of disease progression and the development of associated vascular complications [17].

## Conclusion

Bilateral fibromuscular dysplasia of the internal carotid arteries is an uncommon but clinically important vascular entity that may be overlooked due to its variable and often subtle clinical presentation. This case highlights the pivotal role of imaging in establishing the diagnosis, with characteristic radiologic features—particularly the “string-of-beads” appearance—enabling confident identification. Awareness of this condition is essential among radiologists and clinicians, as early and accurate diagnosis can guide appropriate management, reduce the risk of cerebrovascular complications, and prompt evaluation for multivessel involvement.

## Patient consent

Informed written consent taken from the patient for publication of the case report.
